# Secondary bilateral synchronization of interictal EEG discharges in focal epilepsy: prevalence and associated factors

**DOI:** 10.3389/fneur.2025.1446471

**Published:** 2025-02-04

**Authors:** Polina Specktor, Ronen Spierer, Mark Katson, Moshe Herskovitz

**Affiliations:** ^1^Department of Neurology, Carmel Medical Center, Haifa, Israel; ^2^Rappaport Faculty of Medicine, Technion Institute of Technology, Haifa, Israel; ^3^Department of Neurology, Rambam Health Care Center, Haifa, Israel

**Keywords:** secondary bilateral synchrony, epilepsy, interictal, EEG, epileptiform discharges

## Abstract

**Background:**

Epilepsy is commonly categorized based on etiology, treatment, and prognosis. Misclassification can occur due to the presence of interictal secondary bilateral synchronization (SBS) discharges seen on an electroencephalogram (EEG), misleading the classification process.

**Objective:**

To examine the prevalence of interictal SBS discharges in patients with focal epilepsy and to identify predictors of these discharges.

**Design:**

Retrospective analysis of patients who underwent long-term video EEG monitoring (LTVEM) from August 2001 to May 2014.

**Methods:**

We included patients with focal epilepsy. The patients were divided into two groups based on whether or not they had SBS discharges.

**Results:**

We found 1,017 patients who underwent LTVEM. Of the 221 patients included in the final analysis, 36 (16%) exhibited SBS discharges. Patients in the SBS group were younger and had an earlier onset age of epilepsy. They also had higher rates of unclear seizure onset zone and focal to bilateral tonic–clonic seizures. In the binary logistic regression analysis, young onset age of epilepsy was the only significant factor.

**Conclusion:**

The prevalence of SBS discharges in focal epilepsy is relatively high (16%), emphasizing the importance of cautious interpretation of interictal EEG in epilepsy classification. Young age of disease onset is associated with higher rates of SBS.

## Introduction

Epilepsy is classified as generalized, focal, combined, or unknown. This classification has significance in terms of etiology, treatment, and prognosis (1). Interictal electroencephalogram (EEG) is an essential tool for epilepsy classification; it remains a highly effective and accessible method for demonstrating epileptiform discharges (2). The interictal EEG in generalized epilepsy most commonly shows generalized spike and slow wave discharges, while in focal epilepsy it frequently displays localized discharges, indicative of the seizure’s origin within a specific region of the brain.

As early as 1952, Tükel and Jasper described the concept of epileptiform discharges with secondary bilateral synchronization (SBS) – focal-originated interictal EEG discharges mimicking generalized epileptiform discharges (3). Their initial assumption was that these discharges occur in patients with parasagittal lesions, with peak voltage at the midline or near the midline, but further studies have shown that SBS can result from additional cortical regions (4).

Tükel and Jasper defined SBS as conjugated high-voltage discharges that appear over the homologous area of the epileptic focus at the contralateral hemisphere. This synchrony was later claimed to be mediated through subcortical structures (5) or by intracortical and transcallosal pathways (6).

The existence of interictal SBS discharges can lead to incorrect classification of epilepsy, and over the years we have noted that some patients with SBS were misclassified with generalized epilepsy. In light of this, it is essential to consider the clinical and imaging data of each patient to arrive at a correct classification.

In addition to the problematicity of the diagnosis, Sunwoo et al. found that patients with SBS discharges tended to have poor surgical outcomes with a low post-operative seizure-free rate. They attributed this finding to lesser localization and more rapid spread of the ictal rhythm (7). In their study, which was based on a relatively small sample, the reported prevalence of SBS was 17%. Yet, the literature regarding the prevalence of SBS remains quite sparse. We are aware of two more studies that found SBS present among 5% (8) and 23% (9) of focal epilepsy patients. Moreover, the aforementioned study by Blume and Pillay found SBS present in only 0.5% of patients undergoing EEG (4).

The current study aimed to examine the prevalence of interictal SBS discharges in patients with focal epilepsy and to identify factors that may predict them.

## Methods

### Study participants

This retrospective study included all patients who had focal seizures while undergoing long-term video EEG monitoring (LTVEM) in Rambam Health Care Center between August 2001 and May 2014.

During these years, our unit conducted video monitoring for patients aged 5 and older. However, starting in 2018, with the opening of the pediatric video unit, we transitioned to treating only adults aged 18 and above.

All patients were managed by an expert epileptologist. The diagnosis of focal epilepsy was made in accordance with ILAE guidelines (10), based on appropriate semiology from the patient’s report and video, focal onset on EEG, or a combination of both.

Inclusion criteria for the study included:

1. At least one documented seizure during video EEG monitoring.
2. The seizure was recorded both clinically and electrographically in a clear manner.
3. The patient has focal epilepsy based on subjective and/or objective clinical features and/or focal EEG findings during the seizure.
4. A clear report regarding the interictal EEG was recorded.
5. It is possible to extract demographic information of the patient, including gender, age, and age at disease onset.

Exclusion criteria:

1. Evidence of generalized epilepsy either during the video EEG monitoring or based on medical history.
2. Presence of symptomatic generalized epilepsy

The standard 10–20 system of extracranial electrode placement was used. Anti-seizure medication was usually reduced or completely withdrawn to facilitate the recording of seizures. LTVEM were digitally recorded using a computerized data-acquisition system and later stored on a computer disk. The entire recording for each patient was then visually analyzed by an epileptologist. Analysis included filtering and gain adjustments of suspected epileptiform discharges in bipolar longitudinal, transverse, and ear-referential montages. Only definite spikes, sharp waves, and spike–wave complexes were considered epileptiform abnormalities. If a patient had undergone previous LTVEM, all monitoring results were merged.

Data from patient files was gathered about the following: (1) gender of the patient, (2) age of onset of epilepsy, (3) age at the time of LTVEM performance, (4) referral for LTVEM performance (diagnosis/medical treatment suitability as part of presurgical investigation), (5) previous imaging data (if applicable; only patients with an MRI were included), (6) ictal EEG analysis, including localization and lateralization of the EEG focus and seizure type (focal aware, focal with impaired awareness and focal to bilateral tonic–clonic seizure), and (7) Interictal Epileptiform Discharges (IEDs).

IEDs were further categorized based on the following criteria: localization of the discharges (frontal, temporal, or parietal), lateralization (right or left), and the extent of the discharge (11):

1. Secondary bilateral synchronization (SBS) IEDs: Blume and Pillay (4) define SBS with key characteristics including (a) a lead-in time of at least 2 s, (b) focal triggering spikes that differ distinctly in morphology from bisynchronous epileptiform paroxysms, and (c) a resemblance between the triggering spikes and other focal spikes from the same region. Since these features are not always present (12), and all our patients had confirmed focal epilepsy, any generalized discharges characterized by spike-slow wave or polyspike-slow wave complexes, symmetrically involving both hemispheres, lasting at least 0.5 s, and occurring more than twice during the monitoring period, were classified as SBS.
2. Hemispheric IEDs: Discharges that are widespread across one hemisphere.
3. Multifocal IEDs: Independent discharges occurring in both hemispheres, originating from at least three distinct locations, each separated by more than one interelectrode distance.
4. Bilateral Dependent IEDs: Discharges that appear simultaneously in both hemispheres.
5. Bilateral Independent IEDs: Discharges that appear independently in both hemispheres without synchronization.

**Figure 1:**
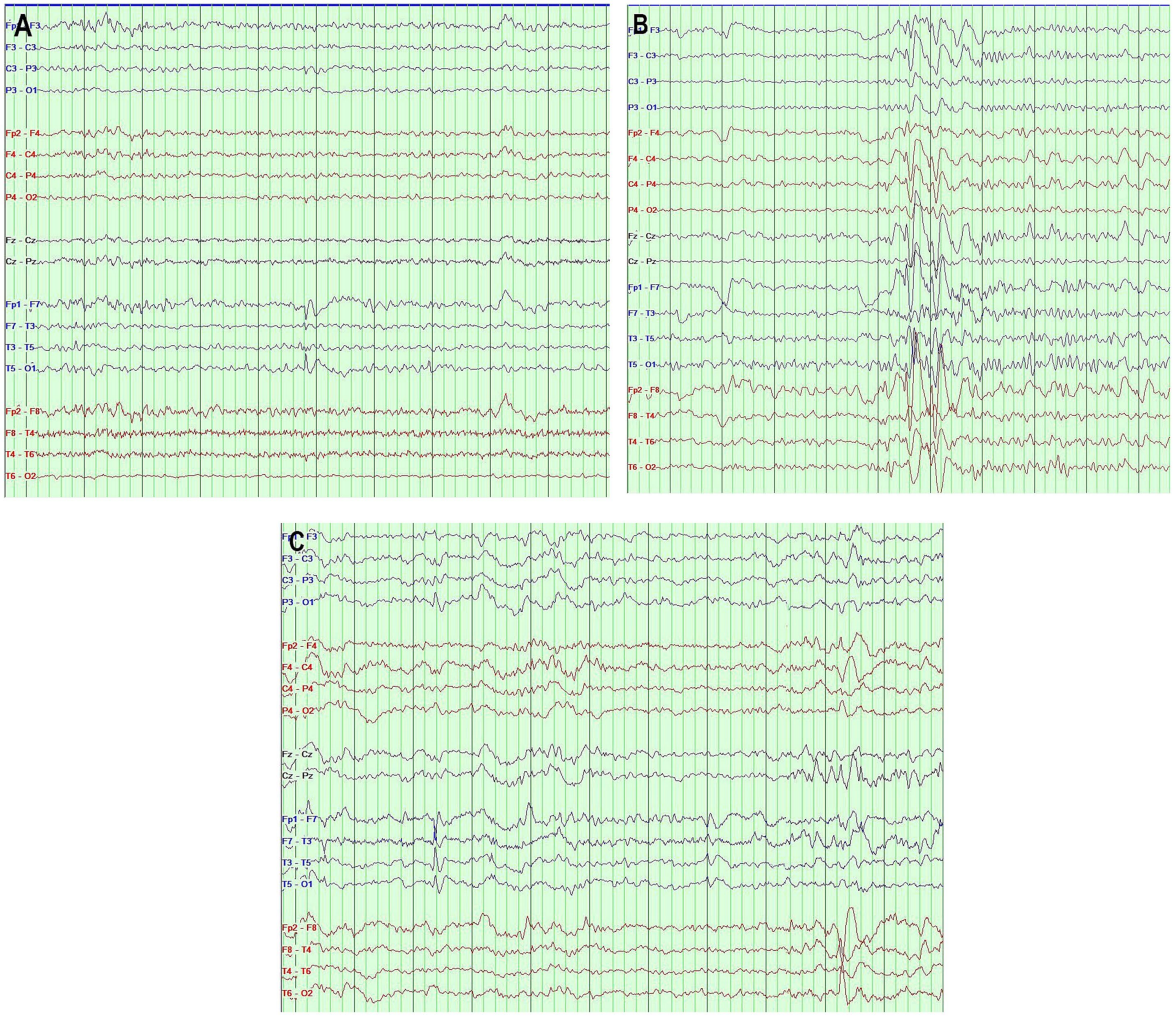
**(A)** Interictal EEG showing sharp waves in the left anterior temporal region. **(B)** Interictal EEG showing secondary bilateral synchronization activity. **(C)** Interictal EEG showing independent bilateral temporal epileptiform discharges. Bipolar montage, page = 10 s, sensitivity 7 μv/mm, filter band pass 0.5–70 Hz (The different EEG segments were taken from three different patients).

Figure 1 illustrates examples of the various types of IED.

### Ethics

All data were gathered in accordance with the declaration of Helsinki. The study was approved by Rambam Health Care Center’s Institutional Review Board.

### Statistical analysis

For the analysis of continuous data, a *t*-test was used. For the analysis of categorical variables, chi-squared and Fisher’s exact tests were used. A binary logistic regression was then used to predict the occurrence of SBS with its possible predictors. All results with *p* < 0.05 were considered significant. Statistical analyses were conducted using IBM’s SPSS 25.0.

## Results

Between August 2001 and May 2014, LTVEM was performed in 1017 patients, resulting in a total of 1,188 reports. The median monitoring time was 4 days. In 241 (24%) patients, focal seizures were recorded.

Twenty recordings had incomplete data: two lacked video descriptions, and two had no ictal data analysis. In four patients, age of epilepsy onset was missing, and in the remaining 12 reports, interictal data was not complete. Therefore, only 221 patients were included in the final analysis.

Reasons for patient referral for LTVEM included the following: confirmation of diagnosis of epilepsy (113 patients, 51%), presurgical monitoring (91 patients, 41%), and epilepsy treatment evaluation (16 patients, 7%).

Ninety-seven patients (44%) were males. Average age of epilepsy onset was 16.11 ± 14.7 years. Average duration of epilepsy at the time of LTVEM was 14.1 ± 12.8 years. Ictal onset was recorded in the following zones: temporal (125 patients, 56.6%), frontal (49 patients, 22.2%), and parietal/occipital (13 patients, 5.9%). In 31 patients (14.0%), the ictal onset zone could not be defined. In 3 patients (1.4%) seizures originated from at least 3 zones. Left hemispheric discharges were recorded in 112 patients (51%) and right hemispheric discharges in 85 patients (38%). The rest of the patients had undetermined or bilateral lateralization sites.

Imaging results were documented in 174 patients: about 38% of the images were normal. The ones that were pathological included mesial temporal sclerosis, tumors, encephalomalacia, cortical dysplasia, periventricular heterotopia, and cavernoma. The rest included inconclusive findings, or rare entities, such as arteriovenous malformation and arachnoid cyst.

IED was recorded in 181 patients (82%) of the 221 patients that underwent final analysis. Regarding the location of IED: 103 patients had pure temporal IED, 12 patients had pure frontal IED, and 3 patients had pure parietal IED. Thirty-four patients had temporal and frontal IED, 13 patients had temporal and parietal IED, 3 patients had temporal, frontal, and parietal IED and 1 patient had frontal and parietal IED. Nine patients had only SBS discharges, 2 patients had only hemispherical IED, and 1 patient had hemispherical and SBS discharges.

Regarding the extent of IED, 36 patients (16%) had SBS discharges. Thirty-one patients (14%) had bilateral dependent interictal activity while 58 patients (26%) had bilateral independent IED. Five patients had multifocal IED. Hemispheric spreading was observed in 10 patients. In 27 patients there was an overlap of SBS discharges with either focal dependent or independent IED. Eighty-eight patients had pure localized one-sided IED.

Regarding the distribution of the interictal SBS discharge: In nine patients, the discharges were maximal across both temporal regions; in three of them, the maximality was on the right, while in the rest, there was no clear lateralization.

In 20 patients, maximal discharges were observed across both frontal regions; in eight, they were maximal on the left, and in two, they were maximal on the right, while in the rest, there was no clear lateralization.

In six patients, maximal discharges were observed across both temporofrontal regions; in two, they were maximal on the left, and in one, they were maximal on the right, while in the rest, there was no clear lateralization.

Additionally, in one patient, maximal discharges were observed across both temporoparietal regions.

Table 1 summarizes the differences between patients with interictal SBS compared to patients without it. Due to a significant difference between the groups for both age and age of onset, a logistic regression was performed to evaluate the individual effect of each variable on bi-synchronization. The model achieved statistical significance: Χ^2^_(2)_=9.15, *p* < 0.01. Yet, onset age was the only significant predictor (*p* < 0.05).

**Table 1:** Differences in the clinical characteristics between SBS and non-SBS patients.

	Sub-variable	Bi-synchronaized (36 patients)	Other (185 patients)	*p*
Age, mean (SD)		25.68 (14.31)	31.46 (16.05)	0.045*
Age of onset, mean (SD)		10.01 (9.70)	17.47 (15.38)	0.001**
Disease duration, mean (SD)		15.09 (15.23)	13.97 (12.42)	0.62
Seizure onset zone, *n* (%)	Occipital	0 (0.0%)	3 (1.6%)	0.001**
	Temporal	12 (33.3%)	113 (61.1%)	
	Unclear	13 (36.1%)	18 (9.7%)	
	Frontal	10 (27.8%)	39 (21.1%)	
	Parietal	1 (2.7%)	9 (4.9%)	
	Multifocal	0 (0.0%)	3 (1.6%)	
Type of seizures, *n* (%)	FAS	8 (22.2%)	44 (23.8%)	0.84
	FIAS	20 (55.6%)	121 (65.4%)	0.26
	FBTC	16 (44.4%)	48 (25.9%)	0.025*
Imaging Findings, *n* (%)	Normal	11 (39.3%)	54 (37.0%)	0.99
	MTS	8 (28.6%)	41 (28.1%)	1.00
	Cortical dysplasia	5 (17.9%)	15 (10.3%)	0.33
	Other#	7 (25.0%)	45 (30.8%)	0.70

*p < 0.05, **p < 0.001.

#Other findings include post-stroke, post-trauma, space-occupying lesions, post-operative changes, cavernomas, and arteriovenous malformation.

SD, standard deviation; FAS, focal aware seizures; FIAS, focal impaired awareness seizures; FBTC, focal to bilateral tonic–clonic; MTS, mesial temporal sclerosis.

## Discussion

The main findings of this study are the relatively high prevalence of SBS and its strong association with early disease onset. While our reported prevalence is similar to the recent study by Sunwoo et al. their results were based on a small sample of patients that underwent surgery (7). Our study had a larger number of participants and was based on all patients admitted to LTVEM. In comparison to a previous large database study, which found SBS present in only 0.5% of patients (4), we found it in 3.5% of all patients that underwent LTVEM. This high prevalence of SBS in focal epilepsy patients shows that caution must be taken in interpreting interictal EEG for epilepsy classification. In addition, it must be remembered that in certain generalized epilepsies, such as juvenile myoclonic epilepsy, we can see the opposite finding related to the fragmentation of the EEG with an impression of focality (13).

As mentioned, SBS was first described by thinking that it is an activity exclusively arising from frontomesial areas. The present work also shows a significant rate of SBS among patients with seizures of temporal origin. Synchrony may arise from epileptic foci of various cortical areas (14) and the ongoing research of the underlying pathophysiology has pointed out different pathways for this phenomenon, such as the corpus callosum and the tapetum (15–17).

The finding of SBS being associated with the onset of epilepsy at a young age is consistent with other studies. Wyllie et al. showed that diffuse IED did not affect the outcome of resective epilepsy surgery. One of the most significant findings in their series was that 90% of lesions occurred during early life. They concluded that diffuse EEG expression is related to the interaction of the epileptic lesion with the developing brain (18). Contrarily, Sunwoo et al. found that SBS was associated with poor surgical outcomes as localization of the lesion is harder (7). Our results are also in agreement with that study, as we found that unclear epileptic focus was more common among the SBS group of patients.

Nonetheless, our results are not consistent with a study by Tinuper et al. In that study, younger age was significantly associated with SBS presence, while young age at disease onset did not reach statistical significance (9). Therefore, further research is needed to confirm our findings and hypothesis.

Admittedly, in this study, the primary goal was to examine the presence of SBS discharges, and therefore, we only partially characterized them. Several points should be emphasized in the reading of interictal EEG: (1) Semiology is of crucial importance in the classification of epilepsy. (2) It must be checked whether there is a lead-in or not. (3) There is a place to check the phase - in their article, Kobayashi et al. concluded that interhemispheric time differences longer than 9 milliseconds are suggestive of SBS (19).

This study has several limitations. One notable limitation is its retrospective design. Additionally, the results are based on patients who underwent LTVEM at a single tertiary center, which may introduce selection bias. The analysis relied on written reports prepared by two senior epileptologists, without an independent review of the EEG recordings. Furthermore, we did not adhere to the original criteria for SBS outlined in Blume and Pillay’s study (4).

It is also possible that some patients in our study have comorbid generalized epilepsy. However, it is important to highlight the fundamental differences between Blume and Pillay’s study and ours. Blume and Pillay’s work (4) focused on interictal EEG data from approximately 10,000 patients, including some without epilepsy. Of these, 76 cases were identified as having suspected SBS, with only 57 meeting his inclusion criteria. Notably, none of the patients underwent video-EEG, making it difficult to accurately assess their epilepsy status. Among these, 49 patients reportedly experienced generalized seizures, with Blume and Pillay suggesting that some were focal seizures evolving into bilateral tonic–clonic seizures. These data indicate that adherence to these criteria likely results in low sensitivity, and unclear specificity.

In contrast, the starting point of our study is patients with confirmed focal epilepsy, as determined by video-EEG. While some patients may have comorbid generalized epilepsy, this does not alter our primary approach. Priority should be given not to interictal discharges observed on EEG alone but to a comprehensive analysis of each case, with particular emphasis on the patient’s medical history. When necessary, detailed etiological and electrophysiological investigations should be conducted to ensure the most accurate diagnosis and the most effective treatment for each patient.

In conclusion, clinicians should be aware of the prevalent phenomenon of SBS which might lead to misclassification when making a diagnosis. As different types of epilepsy need to be treated differently, when interpreting interictal EEG for epilepsy classification, SBS must be considered.

## Data Availability

The raw data supporting the conclusions of this article will be made available by the authors, without undue reservation.
